# The identification of a novel role for BRCA1 in regulating RNA polymerase I transcription

**DOI:** 10.18632/oncotarget.11770

**Published:** 2016-08-31

**Authors:** Rebecca Johnston, Zenobia D'Costa, Swagat Ray, Julia Gorski, D. Paul Harkin, Paul Mullan, Konstantin I. Panov

**Affiliations:** ^1^ School of Biological Sciences, Queen's University Belfast, Belfast, BT9 7BL, UK; ^2^ The Centre for Cancer Research and Cell Biology, Queen's University Belfast, Belfast, BT9 7BL, UK; ^3^ Department of Oncology, University of Oxford, Oxford, OX3 7DQ, UK; ^4^ Krebs Institute, Department of Molecular Biology and Biotechnology, University of Sheffield, Sheffield, S10 2TN, UK

**Keywords:** BRCA1, ribosomal RNA, RNA polymerase I, ribosome biogenesis, cancer

## Abstract

The unrestrained proliferation of cancer cells requires a high level of ribosome biogenesis. The first stage of ribosome biogenesis is the transcription of the large ribosomal RNAs (rRNAs); the structural and functional components of the ribosome. Transcription of rRNA is carried out by RNA polymerase I (Pol-I) and its associated holoenzyme complex.

Here we report that BRCA1, a nuclear phosphoprotein, and a known tumour suppressor involved in variety of cellular processes such as DNA damage response, transcriptional regulation, cell cycle control and ubiquitylation, is associated with rDNA repeats, in particular with the regulatory regions of the rRNA gene.

We demonstrate that BRCA1 interacts directly with the basal Pol-I transcription factors; upstream binding factor (UBF), selectivity factor-1 (SL1) as well as interacting with RNA Pol-I itself. We show that in response to DNA damage, BRCA1 occupancy at the rDNA repeat is decreased and the observed BRCA1 interactions with the Pol-I transcription machinery are weakened.

We propose, therefore, that there is a rDNA associated fraction of BRCA1 involved in DNA damage dependent regulation of Pol-I transcription, regulating the stability and formation of the Pol-I holoenzyme during initiation and/or elongation in response to DNA damage.

## INTRODUCTION

Ribosome biogenesis is a fundamental cellular process that is tightly regulated by an elaborate network of cellular signalling cascades which respond to a variety of intra- and extra-cellular stimuli [1–6]. The synthesis of ribosomal RNA (rRNA) by RNA polymerases I and III (Pol-I & Pol-III) drives ribosome biogenesis and is linked to cell growth and proliferation in eukaryotes [3, 4, 7, 8]. High levels of rRNA synthesis are essential in supporting the unrestrained proliferation of cancer cells, and rRNA transcription is now emerging as a novel target for anticancer therapy [9–13]. In normal cells rRNA synthesis is kept under tight control by various oncogenes and tumour suppressors including p53, Rb, C-MYC, Ppan, CKII and PTEN, and now it is evident that cancer cells have lost some of these restraints [2, 4, 14–17]. It is reasonable to hypothesise that pathways leading to upregulation of ribosome biogenesis are different in different types of malignant cells because different oncogenes and tumour suppressors are affected in different types of cancers.

BRCA1 is 220 kDa nuclear phosphoprotein and known tumour suppressor which is involved in a variety of cellular processes such as DNA damage response, transcriptional regulation, cell cycle control and ubiquitylation [18–21]. Recently, BRCA1 has been shown to play the role of a general repressor of RNA Polymerase III (Pol-III) [22] and in this role it represses transcription of tRNA and snRNA which are required for efficient cell proliferation. BRCA1 selectively regulates transcription of different genes by interacting with variety of polypeptides (for reviews see [23, 24]). *BRCA1* is mutated in approximately 5–10% of hereditary breast cancers [25] and BRCA1 expression is downregulated in up to 40% of sporadic invasive breast carcinomas [26]. Therefore, BRCA1 dysfunction is a significant factor underpinning the development of both hereditary and sporadic breast cancers.

In this study we have investigated the role of BRCA1 in the regulation of transcription of large ribosomal RNAs and selected ribosomal proteins in breast cancer cells. We have shown that BRCA1 is associated with the rDNA repeat and interacts with components of Pol-I transcription machinery. We demonstrate a positive regulatory role of BRCA1 in transcription of rRNA, but found no role for BRCA1 in the regulation of transcription of ribosomal proteins. We found that DNA damage affects both the BRCA1 association with the rDNA and interactions between BRCA1 and Pol-I factors. Together these data suggest that BRCA1 has novel regulatory functions in the control of Pol-I transcription and therefore ribosome biogenesis.

## RESULTS

### BRCA1 associates with rDNA repeat and co-localises with Pol-I

**Figure 1:**
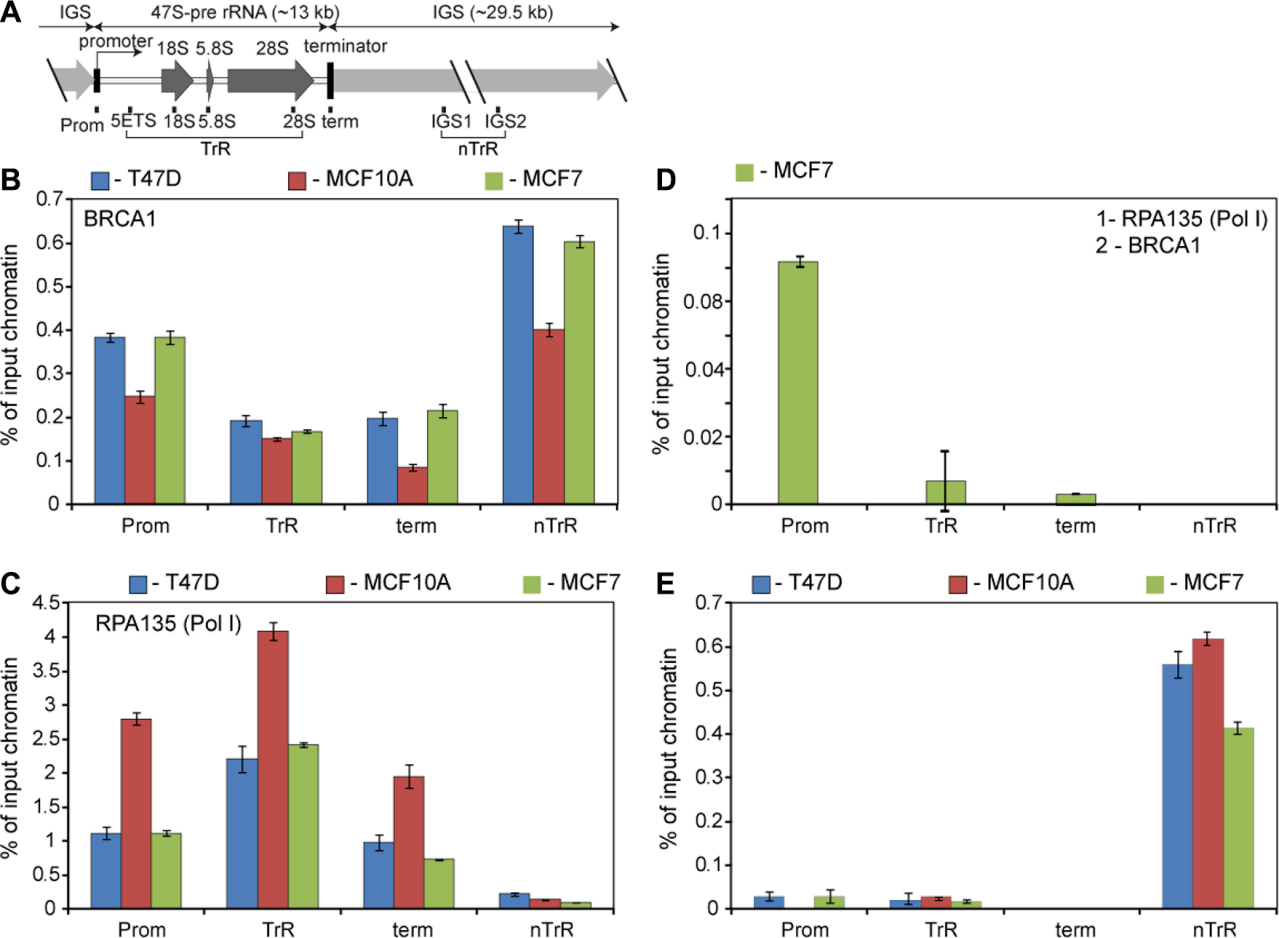
BRCA1 is associated with rDNA repeat (**A**) A diagram of the human rDNA repeat. The positions of eight sets of specific PCR primer/probes used for qPCR analysis of immunoprecipitated DNA are indicated. 5′ETS – 5′-external transcribed spacer; IGS – intergenic spacer; Prom – the rRNA promoter, term – the terminator. Signal representing the transcribed region (TrR) is the average of the combined signal from 5′ETS, 18 S, 5.8 S and 28 S rRNA. Signal representing the non-transcribed region (nTrR) is the average of the combined signal from IGS1 and IGS2. (**B**) ChIP assays were performed using antibodies specific to human BRCA1 and analysed by qPCR using eight sets of specific probes and primers derived from different regions of rDNA repeats (see the diagram above). Internal standards were used for absolute quantification of immunoprecipitated DNA and chromatin input. The value of each bar represents the difference between the signals from the specific antibody and from the negative control (an appropriate IgG) expressed as % from total chromatin input. Signal representing the transcribed region (TrR) is the average of the combined signal from 5′ETS, 18 S, 5.8 S and 28 S rRNA. Signal representing the non-transcribed region (nTrR) is the average of the combined signal from IGS1 and IGS2. The standard deviations from three independent experiments are shown; *n* = 3 (See also Supplementary Figure S8 for raw data). (**C**) ChIP assays were performed using antibodies specific to the second largest subunit (A135) of human Pol-I and analysed as in B. The standard deviations from three independent experiments are shown; *n* = 3. (**D**) Chromatin isolated from untreated MCF7 subjected for the first round of immunoprecipitation using antibody specific to Pol-I subunit A135. After elution chromatin was subjected to the second IP round using antibody specific to human BRCA1 and analysed by qPCR as in B. The standard deviations from three independent experiments are shown; *n* = 3. (**E**) Cells were treated by 5 μM 9HE (Pol I inhibitor) for 1 hour and ChIP assays were performed using antibodies specific to human BRCA1 and analysed as in B. The standard deviations from three independent experiments are shown; *n* = 3.

BRCA1 has been shown to be involved in the regulation of transcription by RNA Polymerases II and III by interacting with transcription factors and regulatory regions of particular genes [22, 27, 28]. We used chromatin immunoprecipitation (ChIP) to examine the association of BRCA1 with various regions of rDNA repeat (Figure 1A). Importantly, antibodies used in this work were previously validated and used in ChIP-seq experiments [29, 30], thus demonstrating sufficient level of specificity.

The level of BRCA1 protein is different in different cells (Supplementary Figure S1), but ChIP results demonstrate similarity in the occupancy levels and in the distribution profile of BRCA1 at rDNA repeat in all cell lines tested (Figure 1B). Consistently BRCA1 is associated with the promoter regions and interestingly a significant fraction of BRCA1 is associated with intergenic spacers (IGS), which contain a number of cryptic promoters and regulatory sequences in addition to various repetitive sequences (i.e. Alu repeats). Notably, IGS is not transcribed by Pol-I, but it is a source of a number of non-coding regulatory RNAs (ncRNAs) transcribed by Pol-II which may be responsible for BRCA1 loading to IGS. Importantly these ncRNAs play an essential role in maintaining the chromatin structure of rDNA repeats and the nucleolus [31, 32].

The level of BRCA1 at the transcribed region is lower, but still significant. Therefore, our results demonstrate that BRCA1 is present at the entire rDNA repeat (∼43 kB), but the occupancy at different regions is not uniform (Pol-I distribution profile in different cell lines is shown in Figure 1C).

Normally, BRCA1 is preferentially associated with promoters, 5′UTR's and exons and significantly underrepresented within introns and intergenic regions [27, 28, 30]. Thus, the BRCA1 distribution profile at rDNA repeat is different from typical BRCA1 profile at the other genomic *loci*. This suggests that BRCA1 fractions associated with different regions of rRNA repeat may play distinct roles which depend not only on the specific region but also on rDNA chromatin structure which is not uniform. More than 300 copies of rDNA repeats are present in human cells and recent studies have revealed that there are three different states of rDNA chromatin: silent, heterochromatic rDNA; poised, transcriptionally competent euchromatic rDNA; and active, transcribed euchromatic rDNA [31, 33, 34]. Results of our sequential-ChIP experiment show that BRCA1 associated only with the promoter of euchromatic repeats (Figure 1D) suggesting that BRCA1 found elsewhere is not involved in transcriptional regulation. Notably, the inhibition of Pol-I transcription by Pol-I inhibitor 9HE [35] lead to selective dissociation of BRCA1 from the promoter (without significant changes in BRCA1 occupancy at the transcribed region and intergenic spacer) also suggesting that association of BRCA1 with promoter region is transcription dependent (Figure 1E).

We examined the subcellular distribution of endogenous BRCA1 in MCF7, MCF10A and T47D cell lines. Immunofluorescence analysis revealed that a small fraction of BRCA1 resides in the nucleoli, and co-localises with Pol-I subunit RPA135 (Supplementary Figure S2A). A significant fraction of BRCA1 is nevertheless localized in the nucleoplasm as it was described elsewhere [36, 37]. Western blot analysis of cytoplasmic and nucleolar fractions showed that presence of BRCA1 in cytoplasm is limited (Supplementary Figure S2B) which is in agreement with the existing data suggesting that BRCA1 cytoplasmic fraction is only increased as result of DNA damage [38–40].

### BRCA1 is not involved in the transcriptional regulation of selected ribosome biogenesis related genes

Regulation of ribosome biogenesis requires coordination of transcription by all three nucleolar polymerases and expression of certain genes. There are examples of proteins (e.g. mTOR) and transcription factors (e.g. C-MYC) which play roles of master coordinators of different stages of ribosome biogenesis [41, 42, 43]. BRCA1 too can affect ribosome biogenesis by regulating transcription of different ribosome components (rRNA, ribosomal proteins) and/or regulatory factors (i.e. components of signalling pathways affecting ribosome biogenesis). It has been shown that BRCA1 is involved in the regulation of at least one component of a ribosome, 5S rRNA [22] and here we show that BRCA1 is involved in the regulation of the synthesis of large rRNAs by Pol-I. Interestingly, the genome wide analysis of BRCA1 binding sites [29] has revealed that at least three genes involved in ribosome biogenesis can be regulated by BRCA1 (Table 1), as it has been found at the promoter area of these genes. To further investigate this, T47D cells were treated with BRCA1 siRNA and HCC1937 cells were transfected with wt BRCA1, and RNA was extracted and converted to cDNA. Expression levels of genes in question were analysed by qPCR. A specific signal was normalised to the signal of housekeeping gene GAPDH and fold of activation or downregulation was determined as ratio between normalised expression level found in treated and the level found in untreated cells. Analysis of the results showed no significant activation or downregulation of expression levels of any of these genes (Table 1). These results suggest that BRCA1 is not involved in the transcriptional regulation of genes in question and therefore, cannot be seen as a general coordinator of ribosome biogenesis.

**Table 1:** Effect of BRCA1 depletion and reconstitution on expression of four genes involved in ribosome biogenesis

Gene	Role	Fold Activation		Significance
		BRCA1 depletion	BRCA1 reconstitution	
**RPL36A**	Ribosomal protein 36, a component of the 60S subunit of the ribosome.	1.03	1.3	No
**RPL12**	Ribosomal protein 12. This protein is a component of the 60S subunit of the ribosome. It is a member of the L11P family of proteins and is found within the cytoplasm.	1.01	1.05	No
**RPS6KB1**	Ribosomal protein belonging to the S6 kinase family of serine/theronine kinases. The protein is activated in response to mTOR pathway leading to increased protein synthesis and cell. proliferation	0.94	0.79	No

Total RNA extracted from treated and control cells was analysed by real-time qPCR using primers specific for each gene including the housekeeper gene GAPDH. Analysis of the qPCR results demonstrated no significant changes (≥ 2 fold) in the relative expression levels of four genes in questions (normalised to GAPDH expression level).

### BRCA1 interacts with Pol-I transcription machinery

BRCA1 interacts with a vast array of proteins forming a variety of different protein complexes with distinct functionality including CTD domain of Pol-II [44] and Pol-III transcription factors Brf1 and Brf2 [22]. One of the known complexes containing BRCA1 is the RNA polymerase II (Pol-II) holoenzyme [45, 46]. This is very large (several MDa) multi-subunit protein complex containing all factors necessary for transcription initiation. The existence of Pol-I holoenzyme has also been suggested previously [47–49] and we performed gel-exclusion chromatography of a nuclease treated T47D nuclear extract using Superose 6 column (exclusion limit over 5 MDa) and analysed a void fraction (containing very high molecular weight protein complexes) by Western blotting. Interestingly, we have detected the presence of BRCA1 and the basal components of Pol-I transcription apparatus (Pol-I, SL1 and UBF) (Supplementary Figure S3).

**Figure 2:**
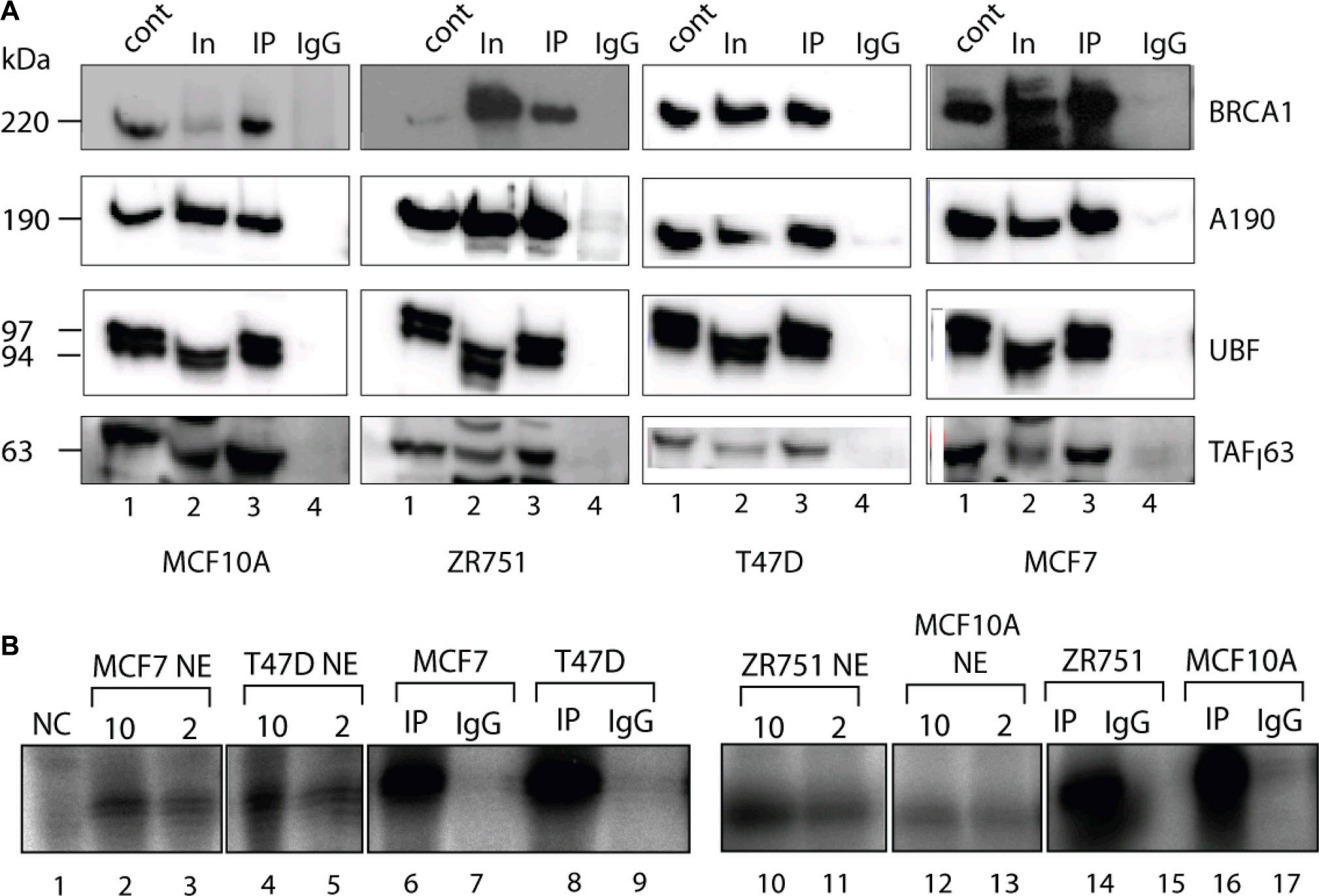
Active components of Pol-I transcription machinery co-immunoprecipitate with BRCA1 (**A)** Nuclear extracts were prepared from actively growing cells and incubated for 4 hours with BRCA1 antibody covalently linked to magnetic beads. The immuno-complexes were washed 4 times by 0.15 M KCl in TM10 buffer and protein complexes were eluted by 8M urea and analysed by Western blotting using antibodies specific to BRCA1, human Pol-I largest subunit A190, UBF and TAF_1_63 subunit of SL1. Lane 1 – HeLa NE (positive control); Lane 2 − input (nuclear extract); Lane 3 – immunoprecipitated complexes; and Lane 4 − negative control (IgG). Positions of prestained molecular weight markers (PageRuller Plus, Fermentas) are indicated. (**B**) Beads carrying immuno-complexes obtained as above were resuspended in transcription mix containing NTP's, rDNA template and Pol-II /Pol-III inhibitor α-amanitine. Transcribed RNA was analysed by S1 nuclease protection assay with a 32P end-labelled DNA oligonucleotide complementary to the first 40 nucleotides of the 5′end of the 47S rRNA primary transcript. Reaction in lane 1 is negative control (2 μl HeLa NE and no template). Reactions in lanes 2, 3, 4, 5, 10, 11, 12 and 13 contain either 10 μl (even numbers) or 2 μl (odd numbers) of nuclear extract isolated from different cell lines as indicated. Reactions in lanes 6, 8, 14 and 16 contain BRCA1 immunoprecipitated complexes and reactions in lanes 7, 9, 15 and 17 contain IgG negative control isolated from different cell lines as indicated.

Next we tested whether BRCA1 interacted with the Pol-I transcription factors by performing co-immunoprecipitation of BRCA1 from nuclease treated nuclear extracts of MCF7, MCF10A, ZR751 and T47D cell lines. Immunoprecipitated complexes were analysed by Western blotting (Figure 2A) and we found that Pol-I, UBF and SL1 all co-immunoprecipitated with BRCA1 in all cell lines, suggesting that BRCA1 either interacts independently with several components of Pol-I apparatus or it is a part of Pol-I holoenzyme.

We have analysed the specific activity of these immunoprecipitated complexes using *in vitro* transcription assays [50] and found that the complexes were able to support specific transcription when supplemented with a template containing the Pol-I promoter (Figure 2B) suggesting that BRCA1 interacts with active basal components of Pol-I apparatus, supporting a holoenzyme hypothesis. None the less, further research are required to characterise BRCA1 interactions with specific components of Pol-I transcription machinery.

### BRCA1 positively regulates rRNA synthesis in cells

The association of BRCA1 with the basal Pol-I machinery and with the rRNA promoter suggest that BRCA1 may be involved in the regulation of Pol-I transcription, similar to its involvement in the regulation of Pol-II and Pol-III transcription [22, 51, 52]. To address a potential role of BRCA1 in the regulation of Pol-I transcription we analysed the effect of BRCA1 depletion and reconstitution on rRNA synthesis.

**Figure 3:**
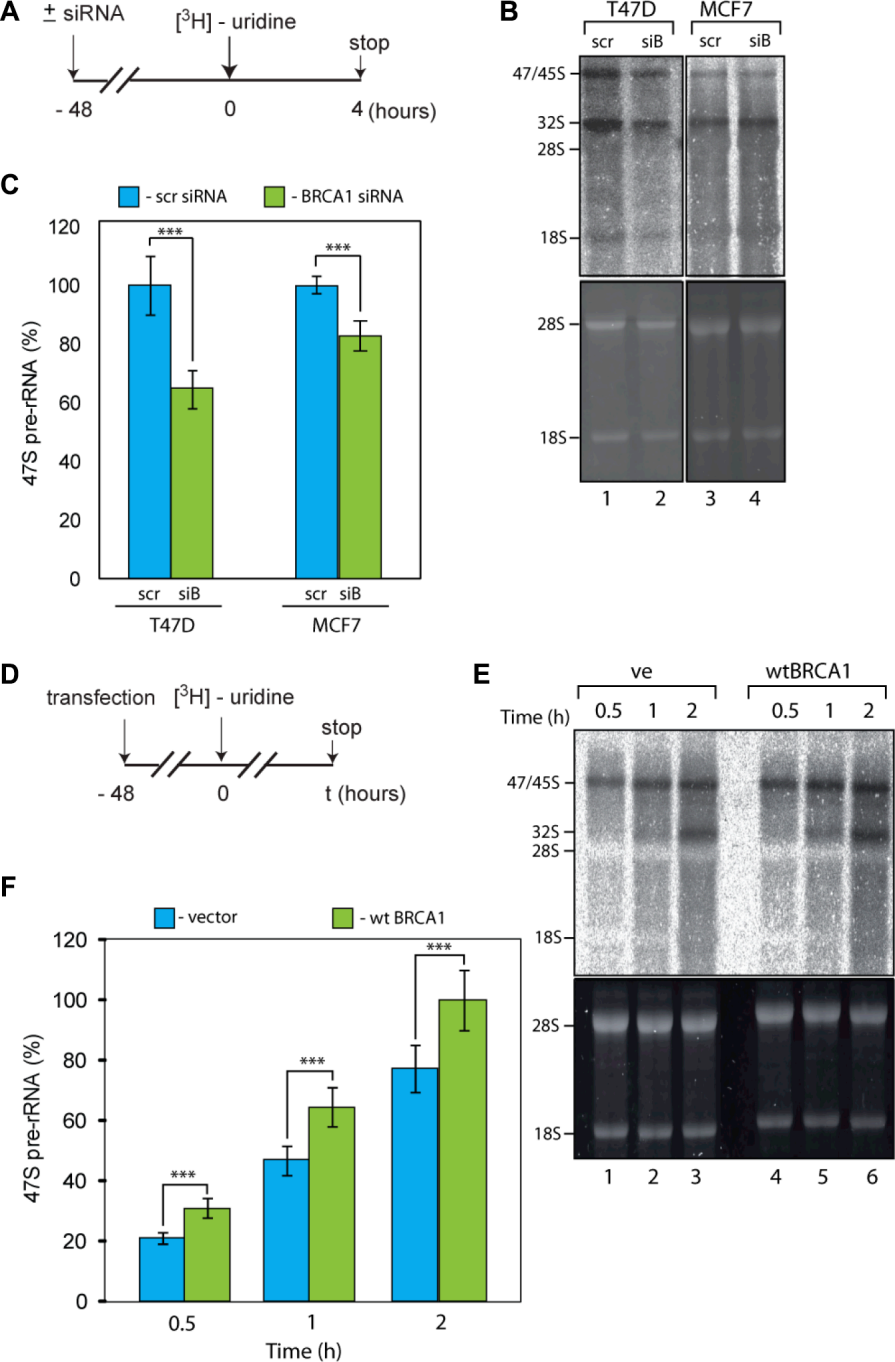
BRCA1 positively regulates rRNA synthesis in cells (**A**) Schematic representation of the labelling of cells with ^3^H-uridine to determine the effect of BRCA1 depletion on ongoing rRNA synthesis. (**B**) RNA was extracted 4 hours after ^3^H-uridine addition and *de nov*o rRNA transcripts were detected by tritium imaging of RNA blots (top panel). Total 18 S and 28 S rRNAs were detected by ethidium bromide staining (bottom panel). (**C**) To determine the relative efficiencies of rRNA synthesis, RNA blots were imaged using tritium image plate (Fuji) and quantitated with the aid of phosphoimager (Fuji) and Aida software (Raytec). Transcript levels are indicated for 47 S pre-rRNA. The data are expressed as a percentage of the highest value (set at 100%). Standard deviations and statistical significance are shown; ***p < 0.001. *P*-values have been calculated using one and two-way ANOVA on R software; *n* = 3. (**D**) Schematic representation of the labelling of cells with ^3^H-uridine to determine the effect of BRCA1 reconstitution on ongoing rRNA synthesis. HCC1937 cells expressing inactive BRCA1 mutant were transfected by construct carrying wt BRCA1. ^3^H-uridine was added 48 hours post-ransfection. (**E**) RNA was extracted 1 and 2 hours after 3H-uridine addition and *de nov*o rRNA transcripts were detected by tritium imaging of RNA blots (top panel). Total 18 S and 28 S rRNAs were detected by ethidium bromide staining (bottom panel). (**F**) To determine the relative efficiencies of rRNA synthesis, RNA blots were imaged using tritium image plate (Fuji) and quantitated with aid of phosphoimager (Fuji) and Aida software (Raytec). Transcript levels are indicated for 47 S pre-rRNA. The data are expressed as a percentage of the highest value (set at 100%); Standard deviations and statistical significance are shown; ***p < 0.001; *P*-values have been calculated using one and two-way ANOVA on R software; *n* = 3.

siRNA mediated depletion of BRCA1 (Supplementary Figure S4A) led to ∼1.8-fold decrease in the synthesis level of the 47S rRNA in T47D cells (Figure 3A, 3B, 3C). The observed effect of BRCA1 depletion in MCF7 cells is more modest (∼20% decrease) but still statistically significant. Importantly, in contrast to the regulation of many other genes a relatively small reduction in the level of rRNA transcription could have a profound effect and maintenance of elevated levels of Pol-I activity in cancer cells appears critically important for cancer cell survival [13]. Therefore our results suggest that BRCA1 depletion negatively affects rRNA transcription and the degree of this effect is different in different cell lines.

To complement these data we next overexpressed exogenous wild-type (wt) BRCA1 in HCC1937 cells which harbour an inactive mutated form of BRCA1. We observed a statistically significant increase in the level of 47S rRNA synthesis in cells expressing wt BRCA1 (Figure 3C, 3D, 3E). Together, all these results suggest that BRCA1 positively regulates rRNA synthesis.

BRCA1 has been shown to be a part of Pol-II holoenzyme [45] and to interact with Pol-III transcription factors [22]. The functional significance of the BRCA1-Pol-II interactions is still unclear, but it has been linked to the BRCA1 surveillance role [44, 46]. In the Pol-III system, interactions between BRCA1 and Pol-III transcription factors play a negative role and BRCA1 represses transcription of Pol-III dependent genes. Here, we found that BRCA1 has a stimulatory role in Pol-I transcription and interacts with the active components of Pol-I pre-initiation complex (PIC). Therefore we can hypothesise that BRCA1 is a part of Pol-I holoenzyme and as such positively affects PIC formation in cycling cells and/or PIC stability, thus stimulating rRNA transcription. It may also have a specific role in stimulating transcription in the context of chromatin regulation. BRCA1 is known to interact with chromatin remodelling complexes including SWI/SNF (for reviews see: [53, 54]), which is known to facilitate Pol-I transcription [55].

**Figure 4:**
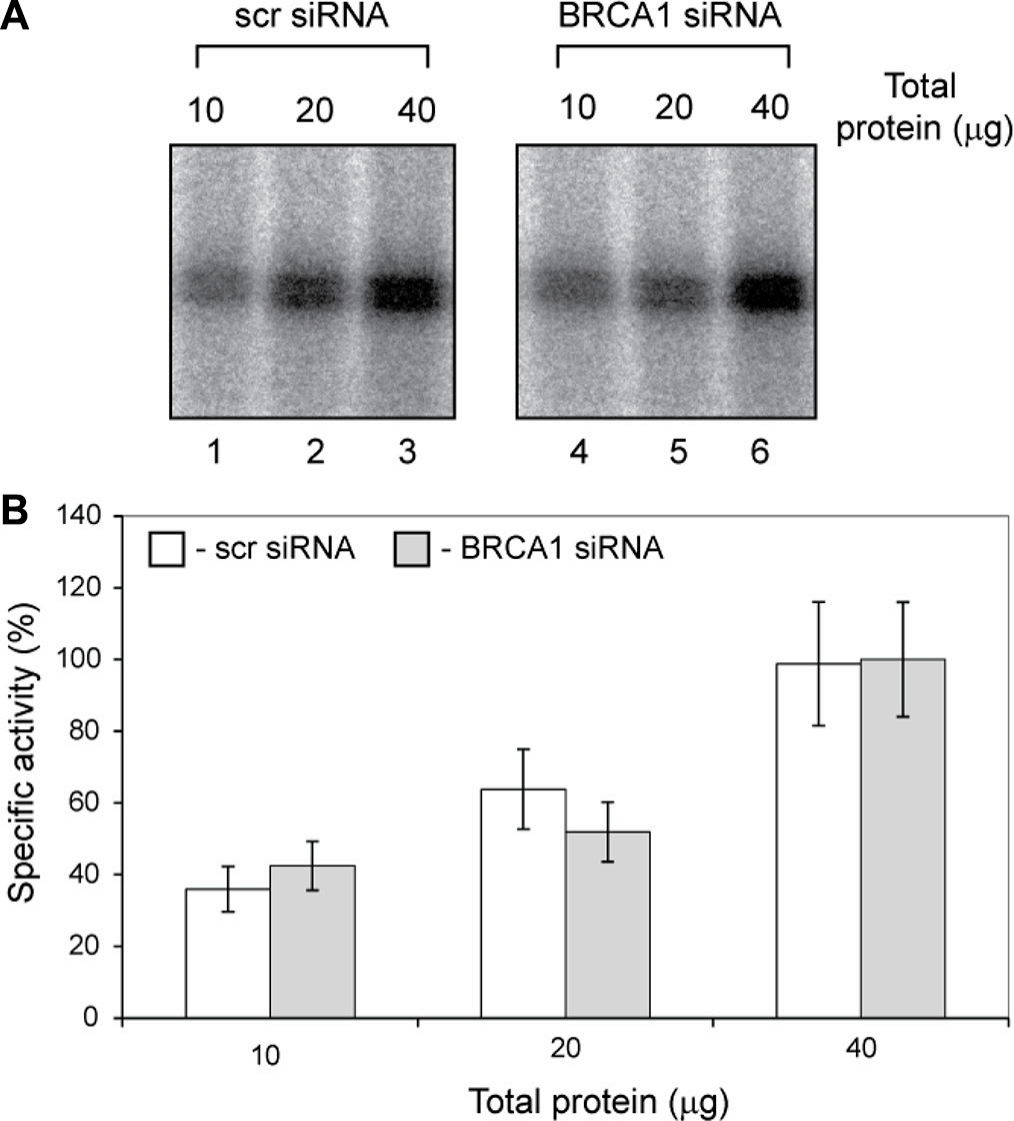
Specific activity of basal Pol-I transcription machinery is not affected by BRCA1 depletion (**A**) Nuclear extracts were prepared from actively growing T47D cells transfected either with scr siRNA or BRCA1 specific siRNA. Specific transcription efficiencies were determined using different quantities of nuclear extracts supplemented with NTP's and rDNA template. Transcribed RNA were analysed by S1 nuclease protection assay with a ^32^P end-labelled DNA oligonucleotide complementary to the first 40 nucleotides of the 5′end of the 47 S rRNA primary transcript. Left panel – cells treated with scr siRNA, Lanes 1, 2 and 3 contains 10, 20 and 40 μg of total protein respectively; Righ panel – cells treated with BRCA1 specific siRNA, Lanes 1, 2 and 3 contains 10, 20 and 40 μg of total protein respectively. (**B**) To determine the relative specific activity signals from panels above were quantitated with aid of phosphoimager (Fuji) and Aida software (Raytec). The data are expressed as a percentage of the highest value (set at 100%); the standard deviations for three independent experiments are shown; *n* = 3.

We have measured specific Pol-I activity in nuclear extracts of untreated and BRCA1 depleted cells using linear, chromatin free DNA template (Figure 4). We were unable to detect any differences in Pol-I specific activity between extracts prepared from BRCA1 expressing and BRCA1 depleted cells. These results strongly suggest that BRCA1 has no direct role in basal Pol-I transcription (in contrast to Pol-III) and supports our hypothesis that BRCA1 stimulates rRNA synthesis in cells via interactions with other factors (i.e. chromatin remodellers or/and chromatin modifying enzymes or transcription activators). Presence of BRCA1 at the transcribed region of rDNA suggest that it can be also part of elongating Pol-I and play a role in transcription-coupled DNA damage response as it was suggested for Pol-II [44].

### DNA damage affects BRCA1 association with rDNA and with Pol-I transcription machinery

After discovering a positive effect of BRCA1 on rRNA transcription we next investigated BRCA1 behaviour at rDNA *loci* in response to various types of DNA damage using a ChIP approach. To determine the optimal dosage of Ultra Violet Radiation (UV)(which causes single-strand breaks) and X-ray (which causes double-strand breaks) radiation we have exposed MCF10A cells to different doses and measured the level of rRNA synthesis (Supplementary Figure S5). We found that 100 J/m^2^ UV treatment lead to 10-fold decrease in Pol-I transcription whereas 50 J/m^2^ had much more modest effect (Supplementary Figure S5B, S5C). Notably, a relatively high X-ray dosage (8 Gy) led to two fold decrease in the level of rRNA synthesis (Supplementary Figure S5D, S5E). The difference in the effect of UV and X-ray radiation most likely is linked to differences in DNA damage pathways activated by different types of DNA damage (for reviews see: [56, 57]) which have different effect on Pol-I transcription [58].

**Figure 5:**
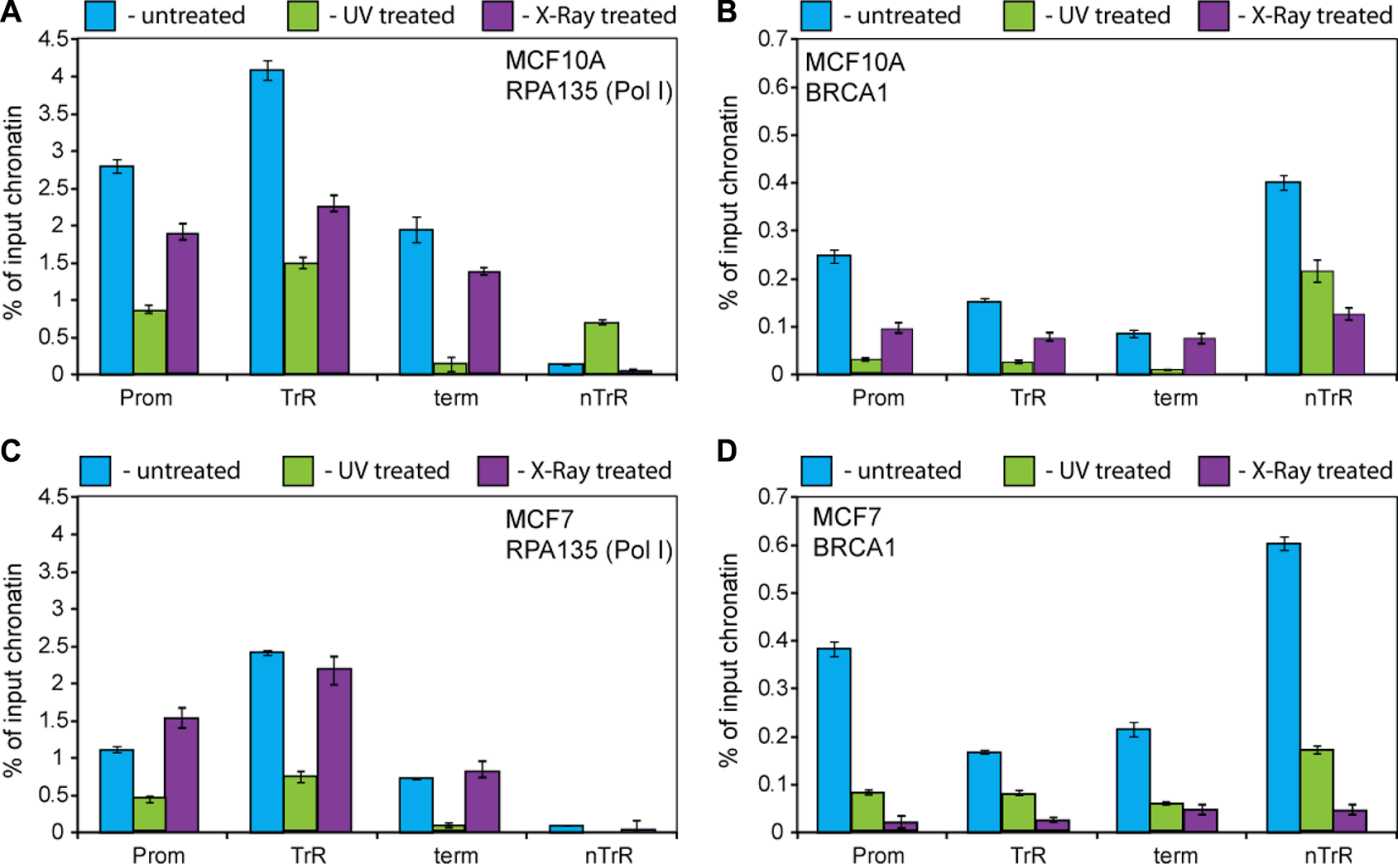
BRCA1 and Pol I association with rDNA affected by DNA damage (**A**–**D**) ChIP assays were performed from chromatin isolated from either UV (100 J/cm^2^) or X-ray (6 Gy) treated or untreated MCF10A and MCF7 cells using antibodies specific either to A135 (A and C) or to human BRCA1 (B and D) as indicated. ChIP DNA was analysed by qPCR. Internal standards were used for absolute quantification of immunoprecipitated DNA and chromatin input. The value of each bar represents the difference between the signals from the specific antibody and from the negative control (an appropriate IgG) expressed as % from total chromatin input. Signal representing the transcribed region (TrR) is the average of the combined signal from 5′ETS, 18 S, 5.8 S and 28 S rRNA. Signal representing the non-transcribed region (nTrR) is the average of the combined signal from IGS1 and IGS2. The standard deviations from three independent experiments are shown; *n* = 3.

BRCA1 is an integral part of various DNA damage response pathways and is directly involved in DNA damage repair. In response to DNA damage BRCA1 accumulates near the damaged *foci* forming part of various DNA repair complexes [59–61]. BRCA1 recruitment is a relatively late event in the complex formation but it is usually completed within 1 hour post-damage [59, 62]. We therefore investigated the recruitment of BRCA1 and Pol-I occupancy at rDNA one hour after UV (100 J/m^2^) or X-ray (6 Gy) treatments in different breast cancer cells (Figure 5). In parallel experiment we also determined effect of irradiation on rRNA synthesis in the same breast cancer cells (Supplementary Figure S6).

We found that UV treatment led to dissociation of BRCA1 from rDNA in MCF10A and MCF7 cells (Figure 5B, 5D). Pol-I (A135) occupancy also decreased following UV treatment (Figure 5A, 5C) which correlates with decreasing Pol-I transcription levels (Supplementary Figure S6A).

X-ray treatment also led to dissociation of BRCA1 from rDNA in all cases (Figure 5B, 5D), however its effect on Pol-I occupancy (Figure 5A, 5C) and rRNA synthesis level (Supplementary Figure S6B) was more diverse. Pol-I occupancy and rRNA synthesis were decreased in MCF10A cells whereas in MCF7 we observed no significant changes in Pol-I occupancy and in rRNA synthesis levels. These data suggest that UV treatment leads to dissociation of Pol-I complexes from rDNA and consequently to significant decreases in transcription levels. In contrast, X-ray treatment leads to the formation of stalled Pol-I complexes (and as a result much smaller changes in Pol-I occupancy) followed by relatively rapid restoration of Pol-I transcription as described earlier [58]. It can be hypothesised that rate of displacement of stalled Pol-I complexes and consequently restoration of transcription is different in different cells which may explain differences observed between MCF10A and MCF7 cells.

**Figure 6:**
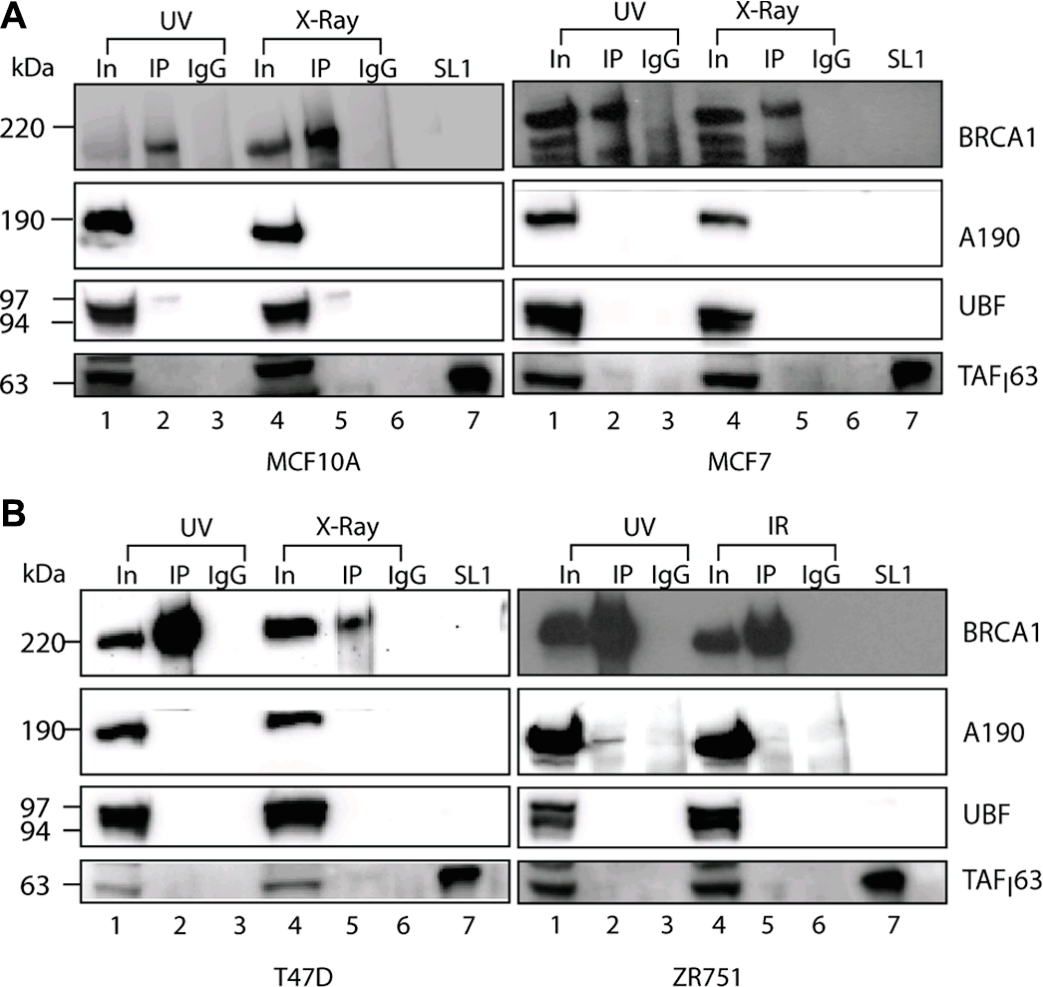
BRCA1 interactions with Pol-I transcriptional machinery affected by DNA damage (**A**–**B**). Nuclear extracts were prepared from either UV (100 J/m^2^) or X-ray (6 Gy) treated cells as indicated. Immuno-precipitation and analysis were performed as in Figure 2A. Lanes 1, 4 − input (nuclear extract). Lanes 2, 5 − immunoprecipitation, lanes 3, 6 − IgG negative control and lanes 7 − purified SL1. Positions of prestained molecular weight markers (PageRuller Plus, Fermentas) are indicated.

We have shown that BRCA1 co-immunoprecipitates with active Pol-I transcription factors from nuclear extracts of untreated breast cancer cells. We next tested if the interactions between BRCA1 and Pol-I machinery were affected by DNA damage by performing BRCA1 imunoprecipitation from nuclear extracts prepared from breast cancer cells one hour after UV (100 J/m^2^) or X-ray (6 Gy) treatments. We found that the interactions between BRCA1 and the Pol-I transcription machinery were disrupted and Pol-I factors no longer co-immunoprecipitated with BRCA1 (Figure 6A, 6B).

## DISCUSSION

BRCA1 is a known tumour suppressor whose main function is to maintain genomic integrity via its critical role in DNA damage repair (for latest review see: [63]) and involvement in the control of a number of fundamental cellular processes such as cell cycle control, transcription, chromatin structure and apoptosis [18–21]. Loss of BRCA1 leads to genomic instability, but the effect of BRCA1 depletion on cell proliferation is more complex and may be positive [64, 65] or negative [66, 67] which is most likely linked to expression of other proteins, or cell type specificities.

In our hands depletion of BRCA1 in all cell lines tested has a negative effect on proliferation (Supplementary Figure S7). We also found that depletion of BRCA1 led to downregulation of rRNA transcription (Figure 3A–3C) whereas introduction of BRCA1 has an opposite effect (Figure 3D–3F). This is interesting and unexpected finding because majority of tumour suppressors involved in the regulation of rRNA synthesis including p53, ARF, Rb and PTEN are known to have inhibitory effect (for latest reviews see [10, 68]). Furthermore, BRCA1 is shown to repress Pol-III directed transcription [22], thus negatively affecting ribosome biogenesis (requiring 5S rRNA and U6 snRNA) and protein biosynthesis (requiring tRNAs). Therefore, we observe an apparent paradox between BRCA1 tumour suppressor role and its functions in Pol-I transcription.

BRCA1 is known to act as a co-activator/co-repressor and its role in regulating Pol-II dependent transcription in general is heavily dependent on its ability to recruit specific transcription factors to relevant promoters. A prime example of this could be the well characterised relationship between BRCA1 and C-MYC, whereby BRCA1 requires C-MYC for recruitment to key target promoters such as hTERT/telomerase [69, 70]. Our colleagues (including several co-authors on this manuscript) have shown that S100 proteins are C-MYC/BRCA1 co-regulated genes in which BRCA1 represses transcription through a C-MYC dependent mechanism and expression of these proteins, most notably S100A7, is strongly upregulated in BRCA1 mutant breast cancer cells [71]. It is well known that C-MYC has an important role in the control of ribosome biogenesis (reviewed in [43]). We propose, in this context that BRCA1 participates in coordinated co-activation of rRNA gene transcription through transcription factors such as C-MYC (and potentially multiple others) probably as a part of Pol-I holoenzyme complex. The role of BRCA1 in Pol-III transcription could be different and better aligned with classical functions of tumour suppressor targeting expression of oncogenes.

Importantly, our findings that basal components of Pol-I transcription machinery co-immunoprecipitate and co-elute with BRCA1 supports this hypothesis. Furthermore, like many BRCA1 interactors, this BRCA1/C-MYC interaction could potentially be altered following BRCA1/Pol-I encountering DNA damage in rDNA. Our observation that interactions of BRCA1 with Pol-I transcription machinery are abolished following DNA damage, supports this hypothesis. In these cases it would be logical to predict that BRCA1 would help stall or remove Pol-I from rDNA promoters to facilitate entry of DNA repair complexes into the damaged region and prevent aberrant transcription. The co-ordinated activation controlled by BRCA1/C-MYC could also go awry in instances of BRCA1 dysfunction (such as in cases of mutation/epigenetic downregulation), resulting in the uncontrolled (C-MYC driven) transcriptional activation of rRNA genes.

Notably, our discovery of BRCA1 interaction with Pol-I machinery is reminiscent of the well described analogous roles for BRCA1 in Transcription Coupled Repair [61, 72]. It is possible that BRCA1 could reside in a complex with Pol-I at rDNA promoters with elongating Pol-I facilitating Pol-I transcription, and in return association with Pol-I would provide BRCA1 with an opportunity to monitor actively transcribed rDNA for DNA damage, including abasic sites, double-strand breaks, intra-strand thymine dimers and inter-strand cross links. Interestingly, different types of DNA damage have different effects on the association of Pol-I with rDNA (Figure 5A, 5C), but similar effects on BRCA1 occupancy (Figure 5B, 5D). This is most likely the consequence of activation of different signalling pathways which either cause rapid dissociation of Pol-I (UV treatment) or accumulation of stalled Pol-I complexes (X-ray) as described earlier [58]. However, in both cases interactions of BRCA1 with Pol-I machinery are weakened (Figure 6) resulting rapid dissociation of BRCA1 from rDNA.

Therefore, interactions of BRCA1 with active Pol-I machinery facilitate a specific targeting of BRCA1 to transcriptionally active rDNA which lead to preferential repair of these rDNA at the expense of non-transcribed repeats. Given large number of transcriptionally inactive rDNA repeats (∼150–300 repeats per human genome) and length of rRNA gene (∼13 kB) this would be especially pertinent. Thus, we propose that stimulatory role of BRCA1 in rRNA transcription is the consequence of BRCA1 surveillance role in rDNA repair.

## MATERIALS AND METHODS

### Tissue culture

MCF7, MCF10A, T47D, ZR751 and HCC1937 cells were obtained from ATCC and maintained according to the supplier's instructions and as described elsewhere. All media were supplemented with 10% FBS (PAA) and 100 U/ml penicillin and 100 μg/ml streptomycin (Gibco). All cells were grown in 37^°C^ incubator at 5% CO_2_.

### Transfections

T47D cells were allowed to grow to a density of 60%. Growth media was removed and replaced with fresh media. A double transfection with 15 nM siRNA (Invitrogen) added 24 hours apart was carried out using RNAi max reagent (Invitrogen) according to manufacturer instructions. Cells were allowed to grow 48 hours postransfection before RNA isolation or metabolic labelling experiments.

Human breast cancer cell line HCC1937 containing germ line mutation in BRCA1 was grown to a density of 70% and transfected with pCDNA3.1 expression plasmid containing wild type BRCA1 or an empty vector using Genejuice (Roche) transfection reagent following manufacturer's instructions in Optimem media. The cells were left 36-72 hours posttransfection before RNA isolation metabolic labelling experiments.

### Antibodies

All antibodies are described in Supplementary Table S1.

### Immunocytochemistry

Cells were grown up to ∼70% confluency (∼50% for starved/re-fed experiments) and were fixed for 10 minutes with 4% paraformaldehyde, permeabilized for 10 min with 1% Triton X-100 and blocked for 10 min with 1% donkey serum in PBS. Cells were then incubated with appropriate antibodies in the blocking buffer (0.1% donkey serum in PBS) for 1 hour, washed 3 × 10 min in PBS and incubated with labelled secondary antibodies for 1 hour. After washes the cells were mounted on a glass slide with Vectashield containing DAPI and visualised using a Leica TCS SP5 confocal microscope.

### Chromatin immunoprecipitation (ChIP) assay

ChIPs were performed essentially as described previously [35, 73]. Briefly, cells were grown until ∼70% confluent, cross-linked with formaldehyde (final concentration 1%) for 10 min and the cross-linking was stopped by addition of glycine (final concentration 0.125 M) for 5 min. Crosslinked chromatin was isolated as described previously [74] and was sheared to 250-base-pair average size. Immunopreciptations were carried out using chromatin isolated from 1 × 10^6^ or 2.5 × 10^5^ cells and appropriate antibodies.

Purified DNA was analysed by qPCR using QuantiFast Multiplex PCR Mix (Qiagen) and two sets (I and II) of four and three primer combinations and probes, which covers different regions of rDNA repeat on Light Cycler 480-II (Roche). PCR parameters and reactions were set as recommended by the PCR Mix manufacturer (Qiagen) and were performed in triplicates, with the standard deviation calculated from three independent ChIP experiments. Results were expressed as percentage of input chromatin and normalized to control IgG levels.

### Immunoprecipitation and immunoblotting

M280 Sheep-anti Mouse beads (Invitrogen) were were washed three times with 0.005% Tween-PBS (PBST). Beads were then incubated with BRCA1 antibody or mouse IgG as a negative control (50 μl:5 μg) for one hour at room temperature followed by 30 minute incubation at room temperature with 5 mg/ml DMP (Pierce) in 0.1 M Borate buffer, pH8.2. The crosslinker is removed and 1 ml 0.1 M Tris-HCL, pH 7.9 was added for 15 minutes. The beads were incubated overnight with 200 μl PBS/0.1%BSA at 4°C. The beads were then washed twice with PBST and twice with 0.1 M KCl in TM10i (TM10i buffer: 50 mM Tris-HCl pH 7.9, 12.5 mM MgCl_2_, 1 mM EDTA, 10% glycerol).

50 μl nuclear extract (5 – 25 mg/ml total protein concentration) prepared as described previously [75] was incubated with 50 μl pre-blocked, crosslinked beads for 4 hours at 4°C. The beads were then placed on a magnet and washed 3 times with 0.1 M KCl in TM10 i buffer and eluted twice, once with 15 μl 8 M Urea followed by 15 μl 2× LDS loading buffer at 37°C for 15 minutes. Eluted proteins were analysed by immunoblotting using various antibodies listed above. The TM10 i buffer was supplemented with EDTA-free complete protease inhibitor cocktail (Roche) and Phosphatase Inhibitor Cocktail 2 (Calbiochem).

### *In vivo* RNA labelling and rRNA analysis

*In vivo* labelling of RNA from cells (60–70% confluent), was performed essentially as described [76], using 10 μCi ^3^H-uridine for ∼0.2–0.4 × 10^5^ cells per well of a 6-well plate. In pulse-chase labelling, cells were incubated for 2 h with ^3^H-uridine, washed and incubated in unlabeled medium containing 0.5 mM uridine. RNA was extracted using Ambion PureLink RNA kit. 2 μg of ^3^H-labelled total RNA was run on a 1% formaldehyde agarose gel at 120 V for 90 min in 1× MOPS running buffer, blotted onto Hybond-N membrane (Amersham), cross-linked (UV-crosslinker, UVP) and analysed by tritium imaging using a Fuji Tritium image plate (or following Perkin-Elmer En^3^Hance spray, exposed to Kodak Biomax XAR film at –80°C), then quantified using Aida software. The standard deviation was calculated from three independent experiments.

### UV and X-ray treatments

Cells were grown in normal growth medium overnight. They were then subjected to 9.9 J/m^2^ in UV crosslinker (UVP) and allowed to grow for 1 hour before harvesting or metabolic labelling experiments.

Cells were grown in normal growth medium overnight. They were then subjected to X-ray treatment of 4 Gy, 6 Gy and 8 Gy. The cells were allowed to grow for 1 hour before harvesting or metabolic labelling experiments.

### Gene expression analysis

RNA from transfected and control cells was purified using the Ambion PureLink RNA kit following the manufacturer's instructions and the RNA concentration was determined spectroscopically. 1 μg of RNA was converted to cDNA using the High Capacity RNA-to-cDNA reverse transcriptase kit (Applied Biosystems). Pol-II transcripts were analysed on the LighCycler 480 thermocycler (Roche) using the Custom gene plate (Roche) according to manufacturer instruction.

## SUPPLEMENTARY MATERIALS FIGURES AND TABLE


