# Supplementary material for: The identification of a novel role for BRCA1 in regulating RNA polymerase I transcription
Source: Oncotarget. 2016 Aug 31;7(42):68097–110. doi: 10.18632/oncotarget.11770 (PMC5356541; doi:10.18632/oncotarget.11770)
Supplement: Supplementary file 1 [file oncotarget-07-68097-s001.pdf]

# The identification of a novel role for BRCA1 in regulating RNA polymerase I transcription

## Supplementary Materials

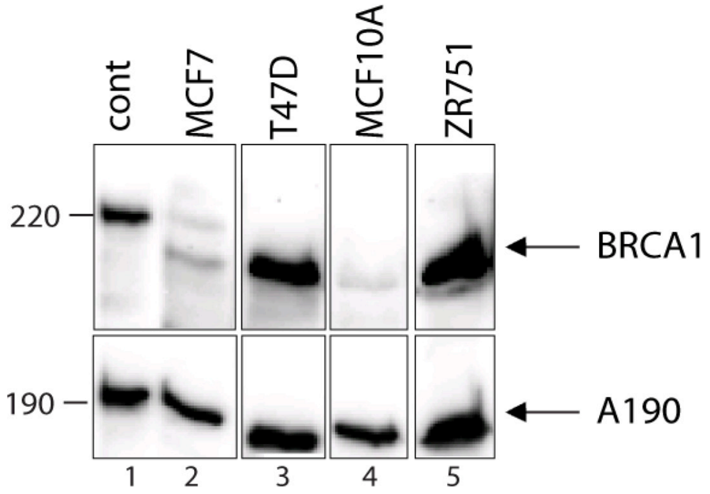


**Supplementary Figure S1: BRCA1 protein level is different in different cells.** Nuclear extracts were isolated from different cell lines as indicated. 50 µg of total protein was analysed by Western blotting using antibodies specific to BRCA1 and human Pol-I largest subunit A190 (used as loading control). Lane 1–HeLa NE (positive control); Lanes 2–5 nuclear extracts prepared from MCF7, T47D, MCF10A and ZR751 cells respectively. Positions of prestained molecular weight markers (PageRuler Plus, Fermentas) are indicated.

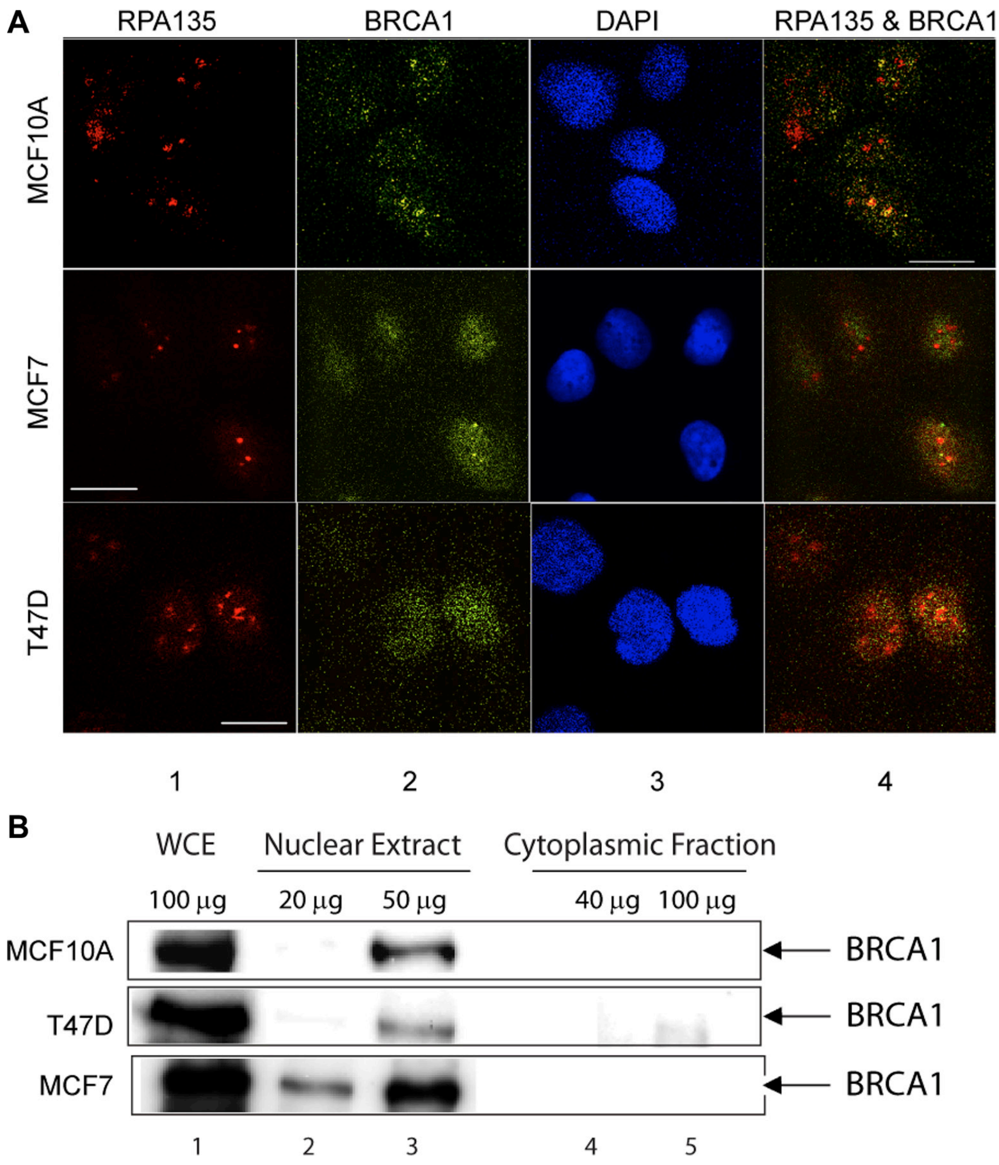


**Supplementary Figure S2: BRCA1 is mainly present in nucleus and its fraction co-localises with Pol-I within nucleolus.** (**A**) Actively growing cells (confluency 60–70%) were fixed following extraction with 1% Triton ×100 and analysed by indirect immunofluorescence using antibodies specific to human Pol-I second largest subunit A135 (panel 1) and human BRCA1 (panel 2). Nuclear DNA was stained by DAPI (panel 3). Merged images (A135 and BRCA1) shown in panel 4. Bar = 5 µM. (**B**) Nuclear, cytoplasmic and whole cell extracts were prepared from different cell lines as indicated. Different quantities of total protein (as indicated) were analysed by Western blotting using antibodies specific to BRCA1 Lane 1 – 100 µg of whole cell extract; Lanes 2, 3–20 and 50 µg protein from nuclear extracts respectively. Lanes 5 and 5–40 and 100 µg protein from cytoplasmic extracts respectively. Positions of prestained molecular weight markers (PageRuler Plus, Fermentas) are indicated.

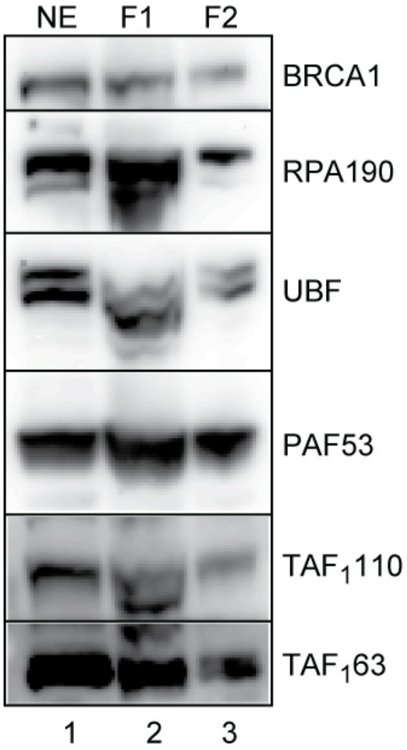


**Supplementary Figure S3: BRCA1 and components of Pol-I transcription machinery are co-elutes in void volume of Superose 6 column.** Total proteins (5 mg) presented in NE prepared from T47D cells were treated with 100 U of Benzonase (Sigma) for 1 hour at 4°C and separated on Superose 6 column (GE Healthcare) equilibrated with TM10/0.15 buffer at flow rate 100 µl/min. Proteins from two void volume fractions (0.5 ml each) were precipitated by TCA and analysed by Western blotting using antibodies specific to BRCA1, human Pol-I largest subunit A190, UBF and TAF 63 subunit of SL1. Lane 1 – 25 µg T47D nuclear extract, Lanes 2 and 3 – void volume fractions.

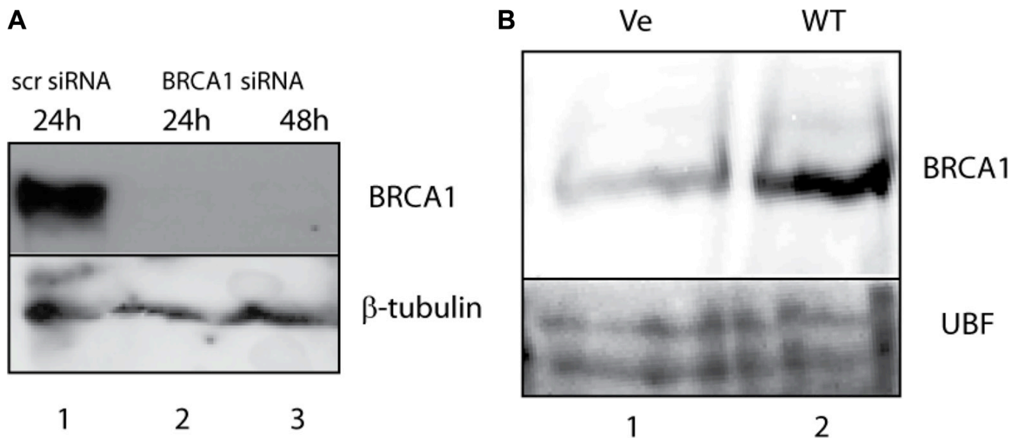


**Supplementary Figure S4: Efficiency of BRCA depletion and reconstitution.** (**A**) T47D cells were transfected with either a non-targeting siRNA (siScr) or a siRNAs directed against BRCA1 and the levels of BRCA1 were determined 24 hours post-transfection by Western-blotting; Lane 1 - non-targeting siRNA, Lanes 2 and 3 – BRCA1 specific siRNA; β-tubulin was used as a loading control. (**B**) HCC1937 cells were transfected either with empty pcDNA3.2 vector (Ve) or with vector carrying wtBRCA1 (WT). The levels of BRCA1 were determined 48 hours post-transfection by Western-blotting; Lane 1 – empty vector, Lane 2– wt expressing construct; UBF was used as a loading control.

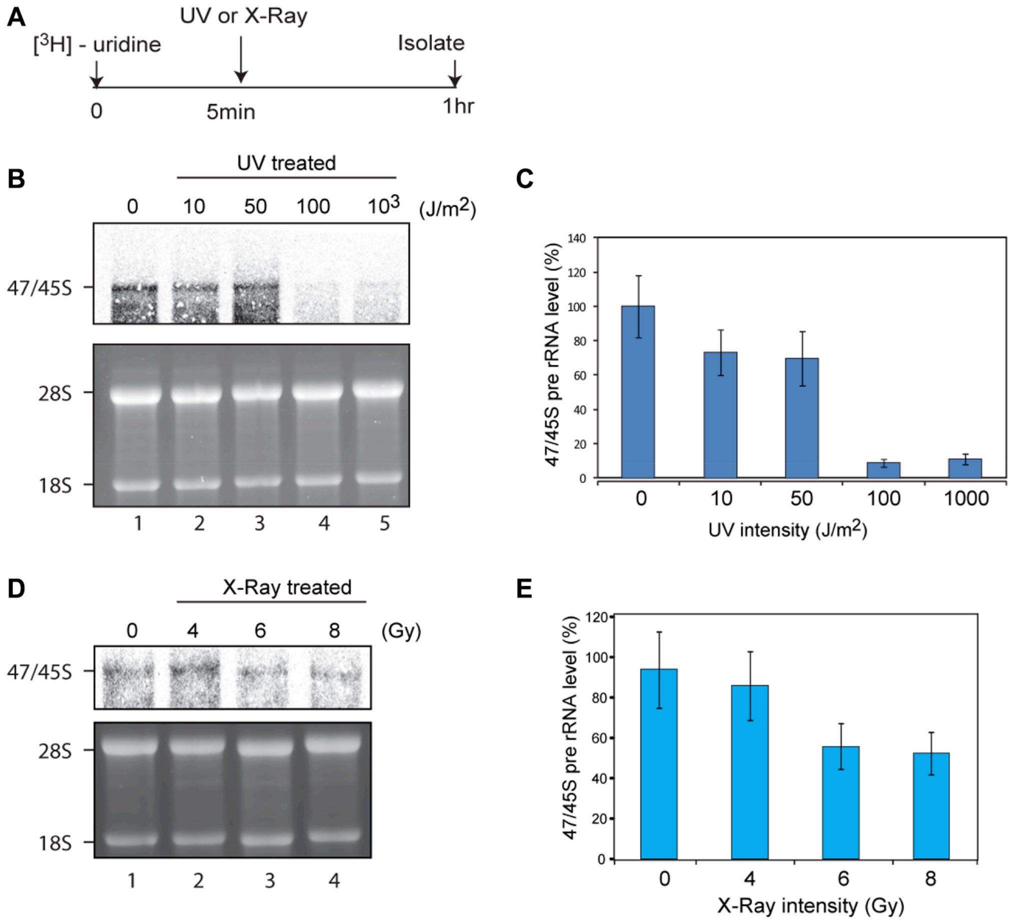


**Supplementary Figure S5: UV and X-Ray treatments affect the efficiency of rRNA synthesis.** (**A**) Schematic representation of the labelling of cells with $^{3}$H-uridine to determine the effect of UV or X-Ray treatments. (**B**) MCF10A cells were treated by different UV dosages as indicated and RNA was extracted 1 hour after UV treatment. *De novo* rRNA transcripts were detected by tritium imaging of RNA blots (top panel). Total 18 S and 28 S rRNAs were detected by ethidium bromide staining (bottom panel). (**C**) To determine the relative efficiencies of rRNA synthesis in UV treated cells, RNA blots were imaged using tritium image plate (Fuji) and quantitated with aid of phosphoimager (Fuji) and Aida software (Raytec). Transcript levels are indicated for 47 S pre-rRNA. The data are expressed as a percentage of the highest value (set at 100%); the standard deviations for three independent experiments are shown. (**D**) MCF10A cells were treated by different X-Ray dosages as indicated and RNA was extracted 1 hour after X-Ray treatment. *De novo* rRNA transcripts were detected by tritium imaging of RNA blots (top panel). Total 18S and 28S rRNAs were detected by ethidium bromide staining (bottom panel). (C, **E**) To determine the relative efficiencies of rRNA synthesis in X-Ray treated cells, RNA blots were imaged using tritium image plate (Fuji) and quantitated with aid of phosphoimager (Fuji) and Aida software (Raytec). Transcript levels are indicated for 47 S pre-rRNA. The data are expressed as a percentage of the highest value (set at 100%); the standard deviations for three independent experiments are shown.

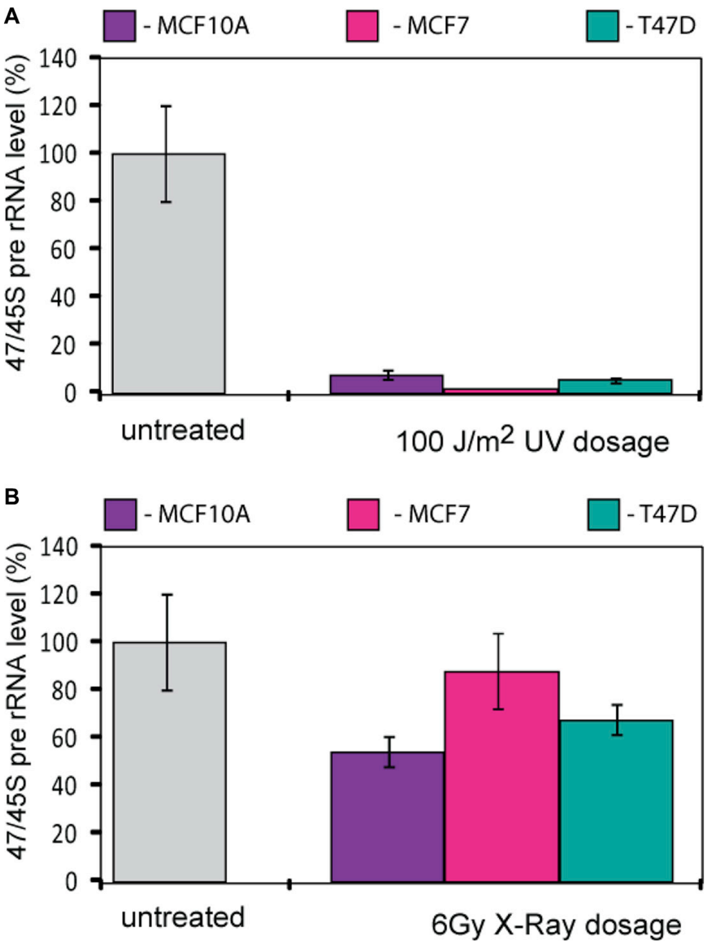


**Supplementary Figure S6: UV and X-Ray treatments affect the efficiency of rRNA synthesis in different cells.** (**A**) $^{3}$H-uridine was added to MCF10A, MCF7 and T47D cells. Cells were irradiated with 100 J/m$^{2}$ of UV. RNA was extracted 1 hour after UV treatment and o rRNA transcripts were detected by tritium imaging of RNA blots. To determine the relative efficiencies of rRNA synthesis in UV treated cells, RNA blots were imaged using tritium image plate (Fuji) and quantitated with aid of phosphoimager (Fuji) and Aida software (Raytec). Transcript levels are indicated for 47 S pre-rRNA. The data are expressed as a percentage of a transcription level in untreated cells (set at 100%); the standard deviations for three independent experiments are shown. (**B**). $^{3}$H-uridine was added to MCF10A, MCF7 and T47D cells. Cells were irradiated with 6 Gy of X-Ray. RNA was extracted 1 hour after X-Ray treatment and o rRNA transcripts were detected by tritium imaging of RNA blots. To determine the relative efficiencies of rRNA synthesis in X-Ray treated cells, RNA blots were imaged using tritium image plate (Fuji) and quantitated with aid of phosphoimager (Fuji) and Aida software (Raytec). Transcript levels are indicated for 47S pre-rRNA. The data are expressed as a percentage of a transcription level in untreated cells (set at 100%); the standard deviations for three independent experiments are shown.

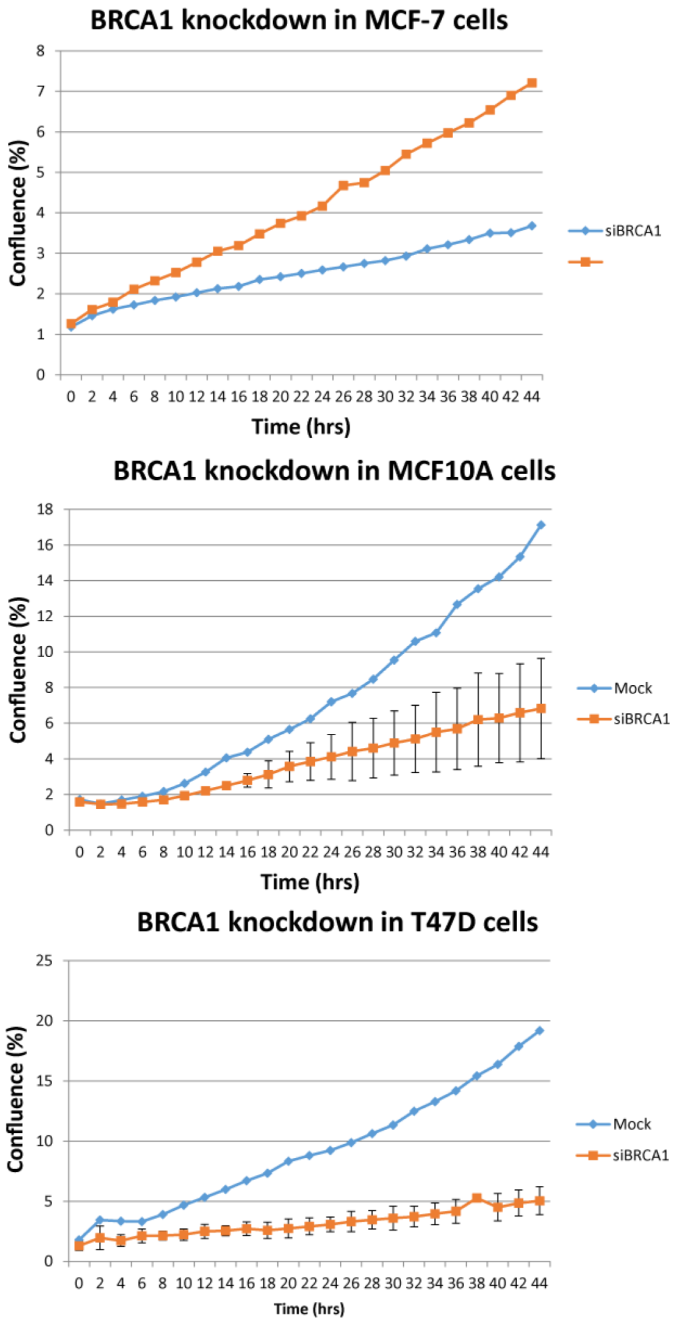


**Supplementary Figure S7: siRNA mediated depletion of BRCA1 lead to negative effect on cell proliferation.** Cells were transfected with either BRCA1 specific siRNA (siBRCA1) or scrambled siRNA (Mock) and 24 hour after transfection re-plated to 96-well plate. The level of confluence of growing cells was measured by IncuCyte (Essen) each hour for 43 hours in total and expressed as %. Mean values for three independent experiments are represented on the graph and standard deviations are shown.

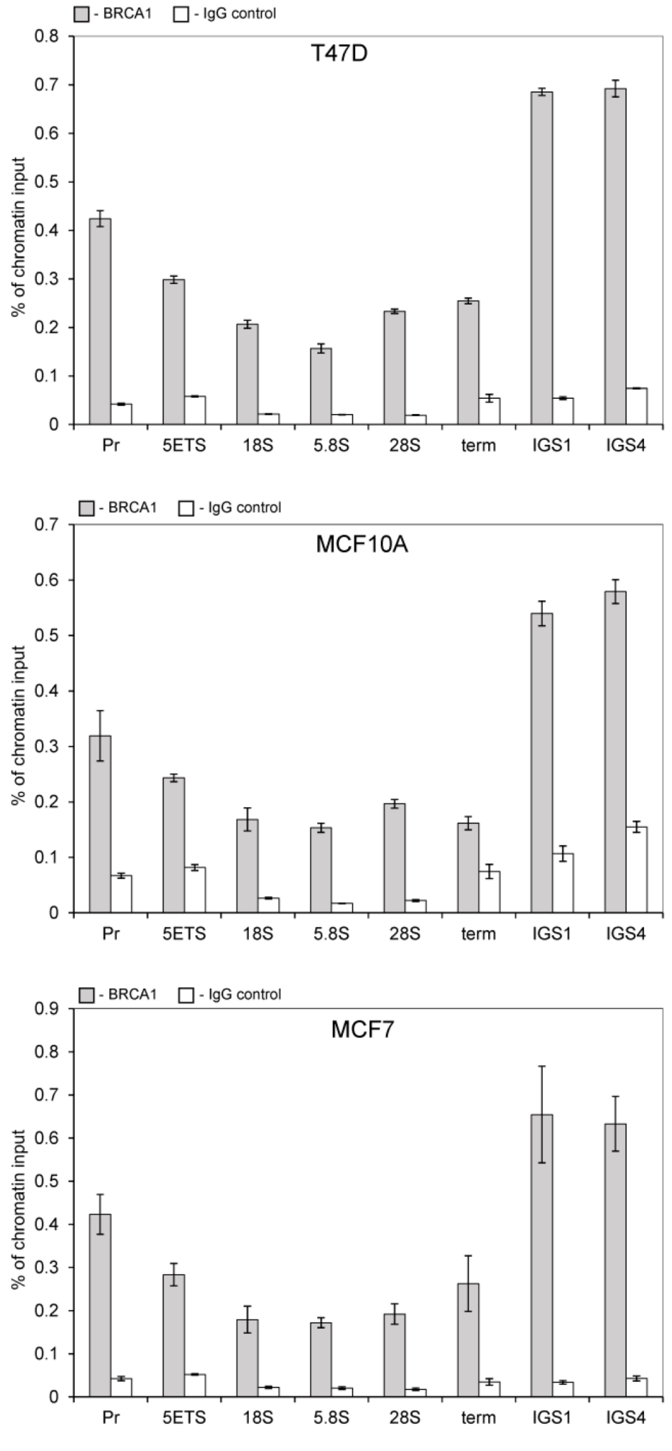


**Supplementary Figure S8: ChIP raw data.** Raw data of ChIP assays performed in Figure 1B. ChIP assay were performed as described in Figure 1B. The value of each bar represents the signal either from the specific antibody or from the negative control (an appropriate IgG) expressed as % from total chromatin input. Signals for all analysed regions (Promoter, 5′ETS, 18 S, 5.8 S, 28 S, terminator, IGS1 and IGS2) are shown. Standard deviations from three independent experiments are shown; $n = 3$.

**Supplementary Table S1: Antibody used in this work**

| Antibody | Origin | Company | Cat.number | WB | ChIP/IP | ICC |
|---|---|---|---|---|---|---|
| BRCA1 (D-9) | Mouse | Santa Cruz | Sc 6954 | | 12.5 µg | 1:50 |
| BRCA1 (AB-1) | Mouse | Calbiochem | OP92 | 1:100 | | 1:50 |
| A194 (C-1) | Mouse | Santa Cruz | Sc 48385 | 1:800 | | 1:100 |
| TAF63 | Rabbit | JZ lab | N/A | 1:1000 | | 1:100 |
| UBF (H300) | Rabbit | Santa Cruz | Sc 9131 | 1:1000 | 12.5 µg | 1:50 |
| Cy3 α-mouse IgG | Donkey | Jackson ImmunoResearch | 715-005-150 | | | 1:250 |
| Cy5 α-sheep IgG | Donkey | Jackson ImmunoResearch | 713-005-147 | | | 1:250 |
| Mouse IgG | Mouse | Sigma | I5381 | | 2.5 µg | |
| Rabbit IgG | Rabbit | Sigma | I5006 | | 2.5 µg | |
| Sheep IgG | Sheep | Sigma | I5131 | | 2.5 µg | |
